# Humans feel too special for machines to score their morals

**DOI:** 10.1093/pnasnexus/pgad179

**Published:** 2023-05-29

**Authors:** Zoe A Purcell, Jean-François Bonnefon

**Affiliations:** Artificial and Natural Intelligence Toulouse Institute, University of Toulouse, 41 All. Jules Guesde, 31000 Toulouse, France; Artificial and Natural Intelligence Toulouse Institute, University of Toulouse, 41 All. Jules Guesde, 31000 Toulouse, France; Toulouse School of Economics and Centre National de la Recherche Scientifique (TSM-R), 1 Esp. de l'Université, 31000 Toulouse, France

**Keywords:** artificial intelligence, morals, psychology, uniqueness, social scoring

## Abstract

Artificial intelligence (AI) can be harnessed to create sophisticated social and moral scoring systems—enabling people and organizations to form judgments of others at scale. However, it also poses significant ethical challenges and is, subsequently, the subject of wide debate. As these technologies are developed and governing bodies face regulatory decisions, it is crucial that we understand the attraction or resistance that people have for AI moral scoring. Across four experiments, we show that the acceptability of moral scoring by AI is related to expectations about the quality of those scores, but that expectations about quality are compromised by people's tendency to see themselves as morally peculiar. We demonstrate that people overestimate the peculiarity of their moral profile, believe that AI will neglect this peculiarity, and resist for this reason the introduction of moral scoring by AI.

**Significance Statement**

The potential use of artificial intelligence (AI) to create sophisticated social and moral scoring systems poses significant ethical challenges. To inform the regulation of this technology, it is critical that we understand the attraction or resistance that people have for AI moral scoring. This project develops that understanding across four empirical studies—demonstrating that people overestimate the peculiarity of their moral profile, believe that AI will neglect this peculiarity, and resist for this reason the introduction of moral scoring by AI.

## Introduction

Morality holds a special role in our personal and social identities (1–5) particularly because it helps us to build the good reputation that is necessary to thrive as a member of a cooperative society (6–11). In small-scale societies, traditional channels like personal experience and gossip can be sufficient to acquire information about the morals of others (12)—but these channels do not scale up well as societies grow, to the point where we can potentially interact with tens or hundreds of thousands of strangers. One radical solution to this scale problem is to delegate the acquisition and collation of moral information to intelligent machines—in other words, to let artificial intelligence (AI) observe the behavior of humans and score the morals of these humans in a way that is intelligible and useful for other humans. We use the term “morals” in a broad sense that includes general moral character, specific moral traits, and moral values. This global approach seems appropriate in the sense that current debates about moral scoring by AI do not distinguish between these different facets of morality. Hence, we speak of AI giving moral scores to individuals in the current broad sense of scoring their general moral character, or their specific moral traits, or the importance they give to some moral values. Here, we show that people are unlikely to accept AI moral scoring, in part because they overestimate the uniqueness of their morals and believe that machines will not adequately take into account this uniqueness.

We are not concerned in this article with well-accepted practices like the aggregation of peer ratings on online platforms. For example, Uber passengers rate the behavior of their drivers (and the other way around), and these ratings are aggregated into a score that is shown to other customers, in order for these customers to anticipate the behavior of their driver (e.g. by gauging their overall likability or acceptability) (13–15). Similar mechanisms exist on other platforms (AirBnB, eBay, etc.), following the same logic: humans rate their experience with another human, and these ratings are aggregated in order for other humans to gain a general impression of each other and predict whether their own experience will be positive or negative (16–18). Taken to the extreme, this peer-rating system could generalize to every interaction, as in the famous Black Mirror episode *Nosedive*, which depicts a world in which people rate every encounter they have with each other. But even in this extreme dystopian version, the scoring is done by humans and not by AI. We are inspired instead by applications where AI observes the online and offline behavioral traces of a human and collates all these traces into one or several moral scores. While much work has been conducted on the way humans judge the morals of machines (19–22), we focus on the novel question of machines that judge the morals of humans.

Building on algorithms that extract personality profiles from online activity (23, 24), there are now AI tools that attempt to automatically score individual social media users on traits such as sexism or aggression (25–27), which can then be used to form a moral impression of these users. AI moral scoring also fuels large-scale social engineering projects such as the Chinese social credit system, in which a wide range of behaviors are aggregated into a single score for every citizen, with social and legal penalties for citizens who drop beneath a certain score. Social credit systems allow governments to define what is moral or immoral behavior, not in any objective sense, but in the practical sense of raising or lowering one's social credit score. Accordingly, they have raised many concerns about abusive social control by authoritarian actors, mischaracterization of individuals, and facilitation of disproportionate social and legal sanctions (28–30). Indeed, based on the recommendation of its expert group on AI to prohibit mass scale scoring of morals (31), the European Commission is considering a ban on social credit systems operated by its member governments (32). Even with such a ban in place, though, there would still be room for less extensive forms of AI moral scoring, deployed by public or private actors (33). For example, people may be willing to let private companies use AI to issue certified scores of their morals, in order to disclose these scores on their CVs or on their dating profiles. As a result, it is important to understand the attraction or resistance that people have for moral scoring by AI, since it will drive their support for AI moral scoring policies and their consumption of AI moral scoring products. We do not know whether AI moral scoring is inevitable, or beneficial on balance, and under which form. We can, however, offer some insights into the psychology of citizens and potential consumers, which will likely shape their reaction to this technology and inform its regulation.

In this article, we seek to show that, reminiscent of the way people resist medical AI because they believe AI cannot grasp the peculiarity of their health status (34), people will resist moral AI because they think AI cannot grasp the peculiarity of their morals. In the medical domain, one's perception of physical uniqueness reduces one's willingness to be diagnosed by AI—we seek to show that in the moral domain, one's perception of moral uniqueness will similarly reduce one's willingness to be scored by AI. For that purpose, we provide empirical evidence for four claims. First, we show that resistance to AI moral scoring is related to its expected accuracy—in other words, that people are less likely to accept AI moral scoring if they expect AI to mischaracterize their morals (claim 1). Second, we show that people overestimate the peculiarity of their moral profile—or more precisely, that they underestimate the prevalence of their moral profile in the population (claim 2). Third, we show that people believe AI moral scoring to be less accurate for peculiar moral profiles (claim 3); and fourth, that people believe as a result that AI is likely to mischaracterize their morals (claim 4). In sum, here we show that people feel too special for AI to score their morals, resulting in resistance to moral scoring by AI.

## Results

### Study 1

To assess claim 1 (*people are less likely to accept AI moral scoring if they expect AI to mischaracterize their morals*), we asked a representative UK sample of 446 participants to rate how acceptable it would be for AI to score each of 17 psychological traits (including 10 moral traits and, for comparison purposes, 4 nonmoral traits, and 3 traits related to mental health; see Materials and methods), and how good AI would be at scoring each of these traits.^1^ Moral and nonmoral traits were distinguished as in previous work (35, 36).

**Fig. 1.**
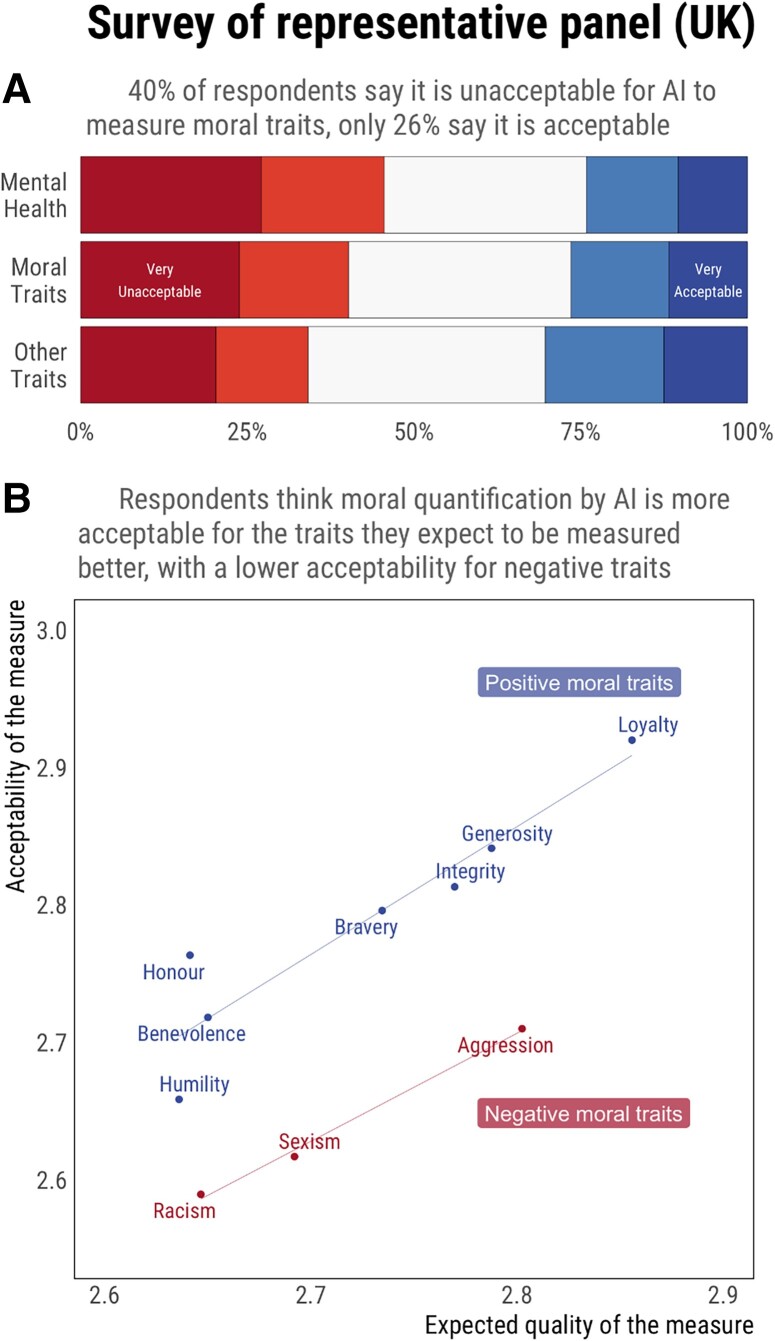
Results from study 1 show that A) participants are more likely to find the scoring of moral traits unacceptable (40%) than acceptable (26%), the five parts of each bar correspond to the proportions of participants responding 1, 2, 3, 4, or 5 to a Likert scale anchored at “very unacceptable” for 1 and “very acceptable” for 5. B) Ratings of acceptability are related to perceptions of the quality of the scoring. The acceptability of scoring negative moral traits is lower than the acceptability of scoring positive traits. More details available in Extended Data Figures: Figure E1.

From a descriptive perspective (see Section 2 and Table S2 of supplementary material for detailed statistics), Fig. 1A shows that 40% of respondents believed it was (very) unacceptable to use AI to measure moral traits compared with 26% who believed it was (very) acceptable. A linear mixed model with a random intercept for participants showed that this acceptability was higher than that of mental health traits (49% believe it is unacceptable versus 24% acceptable) but lower than that of nonmoral traits (35% believe it is unacceptable versus 29% acceptable).

Most importantly, Fig. 1B provides clear visual evidence of a strong positive relationship between expectations about the quality and acceptability of AI moral scoring, together with an acceptability penalty for negative moral traits. A linear mixed model predicting acceptability ratings from expected quality (with trait and participant as random effects) detected an effect of expected quality on acceptability (*B* = 0.31, *P* < 0.001), and all ratings of acceptability and quality were strongly correlated at the trait level (*r* between 0.31 and 0.65, all *P* < 0.001; see Table S2 of supplementary material).

### Studies 2a and 2b

All analyses for studies 2a and 2b were preregistered (https://osf.io/x8rgw).

To assess claim 2 (*people overestimate the peculiarity of their moral profile*), we had to construct participants’ moral profiles, ask them how prevalent they believed their moral profile was, and compare this subjective prevalence to some ground truth about the prevalence of the profile in the population. For this purpose, we used an existing data set from the *Your Morals* project (37, 38) in which 131,015 respondents answered 30 questions about their moral preferences. Using these answers, we categorized the Your Morals respondents into 16 moral profiles corresponding to all combinations of “low” (below median) and “high” (above median) values on four moral dimensions (Care, Fairness, Loyalty, and Authority), and computed the prevalence of each of these 16 profiles.

**Fig. 2.**
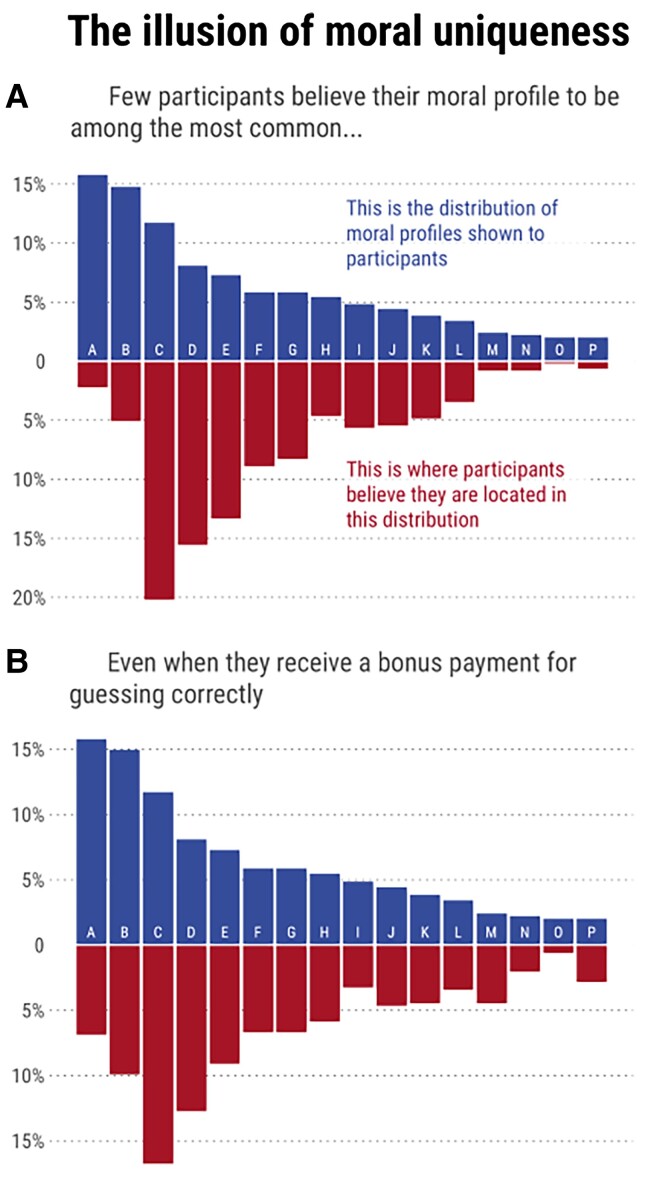
Results from studies 2A and 2B show that participants tend to underestimate the prevalence of their moral profile, when shown an unlabeled histogram of the prevalence of the 16 possible moral profiles (top sections) and asked to guess which bar corresponds to their profile. The red bars display the histogram of guesses by participants. Study 2b offered financial incentives for correct guesses, which did not eliminate the underestimation effect.

Participants in our studies (*N* = 495 in study 2a, *N* = 496 in study 2b) answered the same questions as respondents to the Your Morals project, were categorized into one of the 16 same moral profiles, and shown a summary of their moral profile (for an example, see Fig. S1). Participants were then shown an unlabeled histogram of the prevalence of the 16 profiles (see the top sections of in Fig. 2 A and 2 B) and asked to guess which bar corresponded to their own profile. The only difference between studies 2a and 2b was that their guess was incentivized for accuracy in study 2b but not in study 2a. To assess the extent to which participants overestimated the peculiarity of their moral profile, we compared their guess about its prevalence to its actual prevalence.

Figure 2 displays the guesses (red bars) of participants in study 2a, who were asked which blue bar corresponded to their moral profiles. The distributions of actual and perceived prevalence were markedly different: very few participants believed that their profile was among the most common, and 88% underestimated its prevalence. As preregistered, we fitted a linear model estimating the difference between actual and perceived prevalence that included education, gender, and politics as predictors. The model's intercept was positive and significantly different from zero (2.08, *P* = 0.006), indicating that people underestimated the prevalence of their moral profile. Demographic covariates had no detectable effect on this underestimation (all *P* > 0.40; see Section 3 of supplementary material for detailed results). Study 2b (which offered financial incentives for accurate guesses) delivered very similar findings, as shown in Fig. 2. Once again, the model’s intercept was positive and significantly different from zero (*B* = 5.17, *P* < 0.001), indicating that people underestimated the prevalence of their moral profile. Unlike in study 2a, the model also detected significant effects of demographic variables. The underestimation effect was larger for males (1.45, *P* = 0.040) liberals (1.70, *P* = 0.047; see Section 3 and Tables S3 and S4 of supplementary material for detailed results).

### Study 3

All analyses for study 3 were preregistered (https://osf.io/x8rgw).

We obtained evidence for claim 1 (*people are less likely to accept AI moral scoring if they expect AI to mischaracterize their morals*) in study 1, and for claim 2 (*people overestimate the peculiarity of their moral profile*) in studies 2a and 2b. Study 3 pursues three objectives. First, it provides a preregistered replication of study 1, whose analyses were not originally preregistered (see Section 4 of supplementary material for detailed results). Second, it seeks evidence for claim 3 (*people believe AI moral scoring to be less accurate for peculiar moral profiles*); and third, it seeks evidence for claim 4 (*as a result, people believe that AI is likely to mischaracterize their morals*).

To test claim 3, we asked 506 participants whether AI moral scoring would be more accurate for typical or unique profiles, whether it would make more errors for typical or unique profiles, whether they would trust AI moral scoring more for typical or unique profiles, and whether they would doubt AI moral scoring more for typical or unique profiles. To test claim 4, we asked them how well they thought AI would do at scoring their own moral profile.

**Fig. 3.**
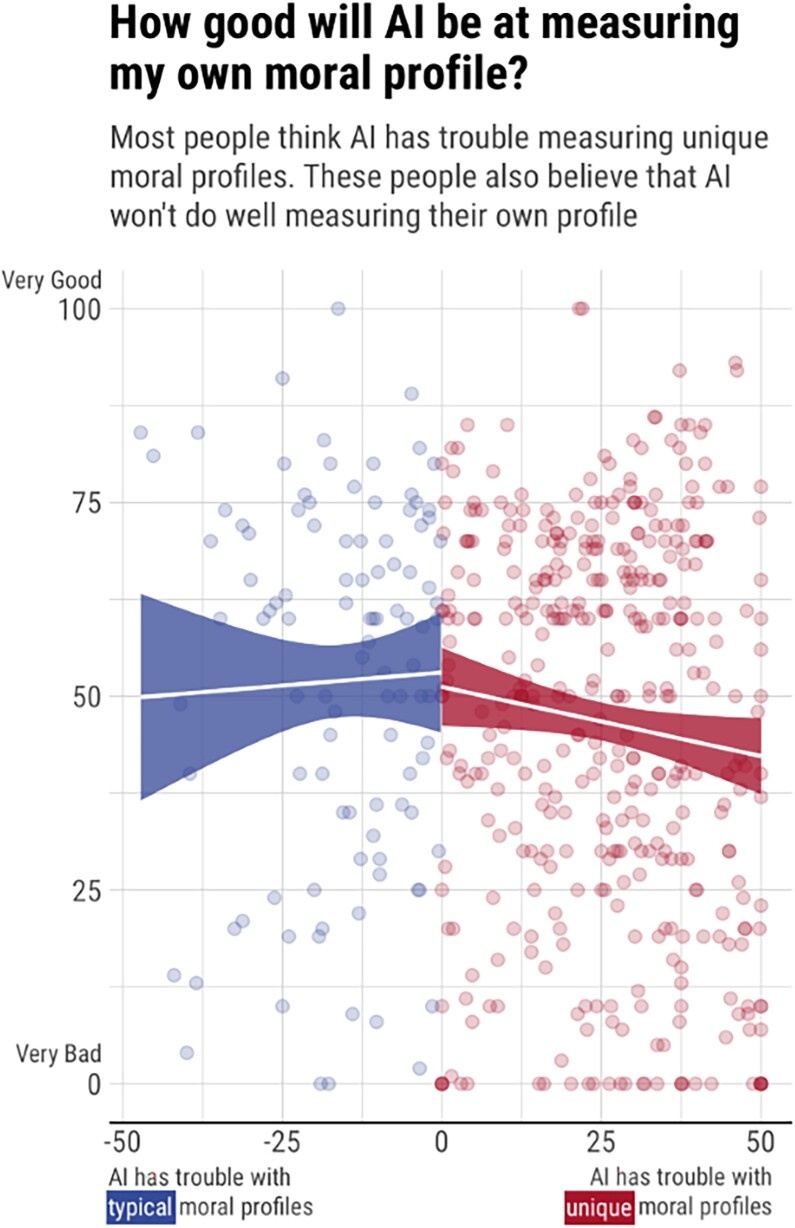
Study 3 showed that many people believe AI will perform more poorly with unique than typical profiles. It also showed that, for these people, the worse they believe AI will be at scoring unique profiles, the worse it will be at scoring their own profile.

Participants’ responses provided clear support for claim 3. Figure 3 displays the distribution of average answers to the four performance questions, and skews toward positive values, that is, in the direction of AI moral scoring having trouble with unique profiles. This was confirmed by an intercept-only linear model, which detected that average answers were significantly greater than zero (intercept = 17, SD = 21; *t*(5,059) = 56.06, *P* < 0.001).

Participants’ responses also provided support for claim 4, with a twist. As we predicted, the more trouble people believed that AI would have scoring unique profiles, the worse they thought AI would do at scoring their own profile (*B* = −0.13, *P* < 0.001). However, visual inspection of Fig. 3 suggests that this association might only be true for people who at least somewhat believe that AI has more trouble with unique profiles (displayed as red dots in Fig. 3), but not true for people who do not hold this belief at all (displayed as blue dots in Fig. 3). Since we did not preregister this prediction, we test it here as an exploratory rather than confirmatory analysis. We fitted two separate linear models testing the blue and red slopes in Fig. 3. The blue slope is not significantly different from zero (*B* = 0.07, *P* = 0.282), but the red slope is, and it is steeper than the slope obtained in the preregistered analysis (*B* = −0.18, *P* < 0.001; see Section 4 of supplementary material for more details).

## Discussion

AI moral scoring offers a scalable solution to the problem of acquiring moral information about strangers in complex societies, but it also comes with ethical risks, giving rise to widespread social concerns and fraught political debates. In this article, we explored the psychological acceptability of AI moral scoring, a factor that will likely play a key role in people's consumption of AI moral scoring services, as well as their demand for AI moral scoring regulations. We showed that the psychological acceptability of AI moral scoring is tightly associated with its expected performance. People will be more likely to accept AI moral scoring if they perceive it as more accurate. This suggests that the acceptability of this technology may increase in the future, provided that its perceived accuracy increases with time. We observed that people found it less acceptable for AI to score negative moral traits (e.g. racism) than positive moral traits (e.g. benevolence), but this may be a simple framing effect: for example, future research may find that people find it more acceptable for AI to score “antiracism” than “racism.” Furthermore, there may be individual and contextual variation in the degree to which some traits, like “aggression,” are perceived as negative.

That said, the psychological acceptability of AI moral scoring may not be ensured by an improvement in its perceived accuracy—because of psychological biases, we documented in this article. We showed that people had a tendency to overestimate the peculiarity of their moral profile, which, in conjunction with their belief that AI would have trouble scoring peculiar moral profiles, led them to doubt that AI would score their morals accurately. This psychological bias compromises the future acceptability of AI moral scoring (which can be good news or bad news, depending on how one feels about the balance of benefits and risks). Even if the average perceived accuracy of AI moral scoring improves with time, people may still feel too special for machines to score their personal morals. In other words, the psychological acceptance of AI moral scoring will unfold based on three factors. First, the perceived accuracy of the technology. Second, the tendency that people have to overestimate the peculiarity of their moral profile. Third, their belief that AI performs less well on peculiar moral profiles.

While perceived accuracy will presumably increase with time, people's overestimation of their moral peculiarity may not, if we assume that this belief is part and parcel of people's general and enduring desire to be (moderately) unique (39). Interestingly, we found in study 2b that left-leaning participants overestimated their moral peculiarity to a larger degree, which aligns with previous research showing that liberals feel a greater need for psychological uniqueness (40)—this suggests that heterogeneity in psychological needs may contribute to some future political polarization about the acceptability of AI moral scoring, in parallel to political disagreements about the social costs and benefits of the technology. Finally, we need to better understand why people believe AI performs poorly when scoring unusual moral profiles, and there is an intriguing parallel here between physical and psychological quantification. Even though people have been exposed to the idea of the quantified physical self for a long time, they still believe that AI is not able to grasp the uniqueness of their physical condition, and thus resist the introduction of AI diagnosis tools (34). This would suggest that even if people are increasingly exposed in the future to the idea of a quantified moral self, they will continue to believe that AI is unable to grasp the uniqueness of their moral profile, and thus resist the introduction of AI moral scoring.

We examined attitudes toward AI scoring of “morals” in a broad sense that includes general moral character, specific moral traits, and moral values. Future research may find it necessary to adopt a higher resolution as the technology and its regulation unfold. For example, it may prove necessary to distinguish between the attitudes people have about AI scoring of the values they hold, and the attitude people have about AI scoring of their overall moral character—or to measure the attitude people have about AI scoring of specific psychological traits, as a function of how well these traits fit into the “moral” category, as well as their perceived variance and base rate in the population. In addition, we were careful not to claim that people resisted moral scoring by AI more than moral scoring by a person. We did not focus on that comparison because moral scoring by AI is mostly relevant in situations where human moral scoring is impossible or impractical, for reasons of scale, making the comparison moot. One possible exception would be a “Nosedive” situation where people rate their interactions with one another, and let machines aggregate the scores. As explained in the introduction, we left these situations outside of the scope of this article, but future research may consider the relative acceptability of moral scoring by AI versus massive peer-to-peer evaluation. Finally, our research investigated abstracted forms of AI moral scoring, rather than focusing on specific contexts such as dating or job applications. This seemed reasonable given the current difficulty of predicting which type of moral scoring will be the first or the fastest to spread, but future research may increase external validity by focusing on a specific application context.

Although digital traces and machine learning have been used to successfully predict demographics like age, gender, and ethnicity, the assessment of moral traits and personal values is evidently more difficult (24, 41, 42). As these technologies improve, however, and their governance is subsequently discussed, it is imperative to understand the psychological drivers of their acceptance. Our findings suggest that there may not be a great appetite for AI moral scoring, even when the technology gets more accurate. While this means that people may approve of strong regulations against AI moral scoring, as discussed by the European Commission, it also means that the commercial potential of this tool might be limited, at least as long as people feel too special for machines to score their morals.

## Materials and methods

### Study 1

#### Participants and design

Study 1 asked a representative UK sample (*N* = 446, *M*_age_ = 47.94, SD_age_ = 15.98, females = 196; see Table S1) how acceptable it would be to score 17 individual traits^2^ related to (i) mental health (e.g. depression), (ii) negative moral (e.g. sexism), (iii) positive moral (e.g. bravery), and (iv) other (e.g. leadership). This allowed us to situate the acceptability of AI-based scoring of moral traits relative to others, and the role of positive versus negative framing. The moral traits included loyalty, generosity, integrity, bravery, honor, benevolence, humility (positive), and aggression, sexism, and racism (negative). The mental health traits included depression, dementia, and social anxiety. Others included extraversion, confidence, orderliness, and leadership. Study 1 was also designed to assess our first claim: that there is a relationship between how acceptable one deems a measure to be and one's expectations for the accuracy of that measure.

#### Procedure

Participants consented to participate and completed brief demographic questions. They then read a short blurb about AI: “In the near future, Artificial Intelligence may be used to measure various personal traits by analysing your digital behaviour and traces. The following questions are designed to gauge whether people believe Artificial Intelligence would be good at performing this task and whether people believe it is acceptable for Artificial Intelligence to perform this task.”

Next, they completed two questions about the acceptability and expected quality of AI-based scoring of 17 personal characteristics (e.g. bravery, depression, leadership):

1. Some people think that using AI to measure personal traits is acceptable, while others think it is not acceptable. Personally, how acceptable do you think it would be to use AI to measure the following traits? (1 = very unacceptable, 5 = very acceptable).
2. Some people think that AI would produce good quality measures of personal traits while others do not. Personally, how good do you think AI would be at measuring the following traits? (1 = very bad, 5 = very good).

### Study 2a

#### Participants

A sample of US participants completed study 2a online, *N* = 495 (*M*_age_ = 25.33, SD_age_ = 7.97, females = 386; see Table S1).

#### Procedure

Participants’ moral profiles were calculated using two established questionnaires from the “Your Morals” project (see yourmorals.org (37, 38), see also (43)). These questionnaires were previously used to describe people's moral preferences and judgments along five dimensions from the Moral Foundations Theory: care, fairness, loyalty, authority, and purity. As in the Your Morals project, participants in the current study completed two 15-item questionnaires. Each questionnaire contained three items about each of the five dimensions. Average scores were calculated per dimension.

The first questionnaire asked, “When you decide whether something is right or wrong, to what extent are the following considerations relevant to your thinking?” An example of an item that loaded onto the authority dimension is: “Whether or not someone showed a lack of respect for authority?” 1 = not at all relevant, 6 = extremely relevant.

The second questionnaire asked, “Please read the following sentences and indicate your level of agreement or disagreement.” An item loading onto the harm dimension is “Compassion for those who are suffering is the most crucial virtue.” 1 = strongly disagree, 6 = strongly agree.

From the participant's responses, we determined their moral profiles using four dimensions: care, fairness, loyalty, and authority. We removed one moral foundation to reduce the number of possible profiles, and opted to remove purity as it has been noted as the least coherent and least clearly moral of the supposed moral foundations (44). For each dimension, participants were categorized as “Low” or “High” if their average score was below or above the population median respectively. For example, participant A could have a profile: “Care: High, Fairness: High, Loyalty: Low, Authority: Low,” and participant B: “Care: Low, Fairness: High, Loyalty: Low, Authority: High,” etc. Population medians were determined by examining a subset of the Your Morals data which includes participants from the United States of America who passed the attention check items and answered all survey items (final *N* = 131,015; data obtained with permission from yourmorals.org).

After completing the two moral preference questionnaires, participants were informed about their moral profiles (for an example, see Fig. S1). They were then presented with an unlabeled plot describing the prevalence of each of the 16 possible profiles and asked “What percentage of people do you think have your profile type? Select the group (bar) that you think your profile belongs to.” Participants responded by selecting 1 of the 16 bars (see the blue distribution in Fig. 2). To provide participants with a reasonable estimate of prevalence rates—the plot reflected the true prevalence rate of all possible profiles in the subset of the Your Morals data described above. Crucially, the plot did not specify which bar reflected which profile. By comparing the prevalence of the participant's moral profile in the obtained data set to the prevalence they selected from the plot, we could assess whether, and to what extent, people believed their moral profile was unique.

### Study 2b

#### Participants

A new sample of US participants completed study 2b online, *N* = 496 (*M*_age_ = 33.47, SD_age_ = 11.42, females = 231; see Table S1).

#### Procedure

Study 2b followed the same procedure as study 2a with the exception that participants were incentivized to give correct responses when selecting the bar that reflected the prevalence of their profile. That is, they were given a bonus payment for a correct choice on the prevalence question.

### Study 3

#### Participants and design

A sample of US participants completed study 3 online, *N* = 506 (*M*_age_ = 33.87, SD_age_ = 12.78, females = 315; see Table S1). To replicate the findings in study 1 and provide participants with more context, participants in study 3 first rated the acceptability and expected quality of ten moral traits (e.g. humility, sexism). They were then presented with a hypothetical moral profile which they were instructed would be based on the relative weight of importance a person might place on these traits (see Fig. S2).

#### Procedure

As in study 1, participants read a short blurb about AI and then responded to one question about acceptability and another about expected quality for 10 moral traits (e.g. humility, generosity).

Participants were then asked to read about moral profiles defined as “… a stable set of moral preferences and judgments. For example, the absolute and relative importance a person places on bravery, humility, generosity, honor, loyalty, benevolence, and integrity.” and provided with an example (see Fig. S2). Additionally, they were instructed “Some profiles are more common than others, we are interested in how you view the relationship between the quality of AI-generated profiles and the prevalence of those profiles. That is, whether you think AI will do a better job at generating accurate profiles for people with common/typical profiles OR for people with rare/unique profiles.”

Participants then responded to four questions about uniqueness neglect. For example, “Do you think the results of a moral profile—generated by Artificial Intelligence—would be more accurate if the profile being assessed was unique or typical? (0 = More accurate for UNIQUE, 100 = More accurate for TYPICAL).” The scale direction was counterbalanced for half of the participants whose scores were subsequently reversed. “Uniqueness neglect” scores were the average of the four items; higher scores reflect greater uniqueness neglect. Finally, participants responded to the question “How good do you think Artificial Intelligence would be at measuring your moral profile? (0 = Very bad, 100 = Very good).”

## Supplementary Material

pgad179_Supplementary_Data

## Data Availability

The data and analysis code are available at https://osf.io/x8rgw.

## Footnotes

1 This work was exploratory and not preregistered. It formed a part of an omnibus survey.

2 These traits were selected on the basis that they are easily understood by the general population (i.e. compared with more complex moral characteristics like utilitarianism) and could be easily translated for future studies with non-English speakers.
