# Correction to: Humans feel too special for machines to score their morals

**DOI:** 10.1093/pnasnexus/pgad379

**Published:** 2023-11-15

This is a correction to: Zoe A Purcell, Jean-François Bonnefon, Humans feel too special for machines to score their morals, *PNAS Nexus*, Volume 2, Issue 6, June 2023, pgad179, https://doi.org/10.1093/pnasnexus/pgad179

During a retroactive audit conducted by *PNAS Nexus*, it was discovered that this paper was missing a statement acknowledging compliance with the *PNAS Nexus* Human and Animal Participants and Clinical Trials policy:

All studies in this paper were approved though the TSE-IAST Review Board for Ethical Standards in Research under the code 2021-03-001. In each study, all participants consented to participate.

This error has been corrected in the original article.

