# Supplementary material for: Humans feel too special for machines to score their morals
Source: PNAS Nexus. 2023 May 29;2(6):pgad179. doi: 10.1093/pnasnexus/pgad179 (PMC10266524; doi:10.1093/pnasnexus/pgad179)
Supplement: pgad179_Supplementary_Data [file pgad179_supplementary_data.zip › PNASNEXUS-PNASNEXUS-2022-01256R-s03.docx]

### SUPPLEMENTARY INFORMATION

*Section 1. Extended Information About the Samples*

Table S1. Extended Information About the Samples

*Information about the demographic variation for each of the four studies.*

| Study (N) | Gender | Age  M (SD) | Education | Politics |
| --- | --- | --- | --- | --- |
| 1(446) | Female: 196 Male: 241 Non-binary/third gender: 4 Prefer not to answer: 2 | 47.94 (15.98) | No completed education: 16 Primary education: 7 Lower secondary education: 78 Upper secondary education: 132 Post-secondary including pre-vocational or vocational education but not tertiary: 71 Tertiary education (advanced): 85  Tertiary education (first level):56 | Not available. |
| 2a (495) | Female: 386 Male: 92 Non-binary/third gender: 17 Prefer not to answer: 0 | 25.33 (7.97) | No high school degree*: 3 High school diploma: 71 Some college: 152 College/other tertiary degrees: 269 | Conservative: 53 Liberal: 312 Moderate: 108 Other: 22 |
| 2b (496) | Female: 231 Male: 252 Non-binary/third gender: 13 Prefer not to answer: 0 | 33.47 (11.42) | No high school degree*: 5 High school diploma: 44 Some college: 152 College/other tertiary degrees: 356 | Conservative: 115 Liberal: 254 Moderate: 109 Other: 18 |
| 3 | Female: 315 Male: 175 Non-binary/third gender: 13 Prefer not to answer: 3 | 33.87 (12.78) | No high school degree*: 3  High school diploma: 48 Some college: 131 College/other tertiary degrees: 324 | Conservative: 73 Liberal: 285 Moderate: 122 Other: 26 |

Note. **Because there were so few participants in this group, we combined them with the nearest category for analyses.*

*Section 2. Extended Information about Study 1 Results*

*Ratings of Acceptability*

To examine the public acceptance of AI-based scoring of moral traits relative to others, we categorized traits as 1) mental health, 2) negative moral, 3) positive moral, and 4) other. The mean ratings of acceptance were highest for other traits such as leadership (eM=2.87, SE=.05), followed by positive moral traits (eM=2.76, SE=.05), negative moral traits (eM=2.65, SE=.05), and finally mental health traits (eM=2.62, SE=.05). The mean ratings of expected quality were highest for other traits such as leadership (eM=2.97, SE=.04), followed by positive moral traits (eM=2.70, SE=.04) and negative moral traits (eM=2.70, SE=.05), and finally mental health traits (eM=2.58, SE=.05).

To formally analyze whether the trait category predicted ratings of acceptability and quality we fitted linear mixed models (estimated using REML and nloptwrap optimizer) to predict rating with category (formula: rating ~ 1 + category) with participant included as a random effect. To contain the number of follow up comparisons, we only examined those including moral traits (positive or negative) as a reference group.

For acceptability with positive moral traits as the reference group, the model's intercept corresponding to positive moral traits was at 2.76 (95% CI [2.66, 2.86], t(7576) = 53.58, p < .001). The effect of category [negative moral traits] was statistically significant and negative (beta = -0.11, 95% CI [-0.16, -0.07], t(7576) = -4.71, p < .001; Std. beta = -0.09, 95% CI [-0.12, -0.05]). The effect of category [mental health traits] was statistically significant and negative (beta = -0.14, 95% CI [-0.19, -0.09], t(7576) = -5.81, p < .001; Std. beta = -0.11, 95% CI [-0.14, -0.07]). The effect of category [other traits] is statistically significant and positive (beta = 0.11, 95% CI [0.06, 0.15], t(7576) = 4.95, p < .001; Std. beta = 0.08, 95% CI [0.05, 0.12]).

When using negative moral traits as a reference group, the model's intercept, corresponding to moral negative traits, was at 2.65 (95% CI [2.55, 2.76], t(7576) = 49.34, p < .001).The effect of category [mental health traits] was statistically non-significant and negative (beta = -0.03, 95% CI [-0.08, 0.03], t(7576) = -0.93, p = 0.354; Std. beta = -0.02, 95% CI [-0.06, 0.02]). The effect of category [other traits] is statistically significant and positive (beta = 0.22, 95% CI [0.17, 0.27], t(7576) = 8.32, p < .001; Std. beta = 0.17, 95% CI [0.13, 0.21]).

These acceptability results show that AI-based scoring of positive moral traits was rated as less acceptable than that for other traits but more acceptable than that of negative moral traits and mental health traits. Additionally, the acceptability of AI-based scoring of negative moral traits was rated less acceptable than that for other traits but no different to that of mental health traits.

*Expected Quality*

For expected quality, with positive moral traits as the reference group, the model's intercept, corresponding to positive moral traits, was at 2.70 (95% CI [2.62, 2.78], t(7576) = 62.43, p < .001). The effect of category [negative moral traits] was statistically non-significant and positive (beta = 1.17e-03, 95% CI [-0.05, 0.05], t(7576) = 0.04, p = 0.965; Std. beta = 9.85e-04, 95% CI [-0.04, 0.04]). The effect of category [mental health traits] is statistically significant and negative (beta = -0.12, 95% CI [-0.17, -0.07], t(7576) = -4.41, p < .001; Std. beta = -0.10, 95% CI [-0.14, -0.05]). The effect of category [other traits] is statistically significant and positive (beta = 0.27, 95% CI [0.22, 0.32], t(7576) = 11.05, p < .001; Std. beta = 0.22, 95% CI [0.18, 0.26]).

When using negative moral traits as a reference group, the model's intercept, corresponding to moral negative traits, was at 2.70 (95% CI [2.61, 2.79], t(7576) = 58.19, p < .001). Within this model: The effect of category [positive moral traits] is statistically non-significant and negative (beta = -1.17e-03, 95% CI [-0.05, 0.05], t(7576) = -0.04, p = 0.965; Std. beta = -9.85e-04, 95% CI [-0.04, 0.04]). The effect of category [mental health traits] is statistically significant and negative (beta = -0.12, 95% CI [-0.18, -0.06], t(7576) = -3.77, p < .001; Std. beta = -0.10, 95% CI [-0.15, -0.05]). The effect of category [other traits] is statistically significant and positive (beta = 0.27, 95% CI [0.21, 0.32], t(7576) = 9.03, p < .001; Std. beta = 0.22, 95% CI [0.17, 0.27]).

These results show that the ratings of expected quality for AI-based scoring of positive moral traits were no different from that for negative moral traits. AI-based scoring of both positive and negative moral traits was rated as higher quality than that of mental health traits and lower quality than that of other traits.

*The relationship between Expectations of Quality and Acceptability*

As expected, all ratings of acceptability and quality were strongly and significantly related at the trait level (r between .31 and .65, p<.001; see S2 Table 2). We fitted a linear mixed model (estimated using REML and nloptwrap optimizer) to predict acceptability ratings with quality ratings (formula: ‘acceptability rating’ ~ ‘quality rating’). The model included trait (e.g., extraversion) and participant as random effects. The effect of quality rating was statistically significant and positive (beta = 0.31, 95% CI [0.29, 0.33], t(7577) = 31.52, p < .001; Std. beta = 0.29, 95% CI [0.27, 0.31]).

Table S2. Correlations between ratings of acceptability and expected quality at the trait level.

*Correlations between ratings of expected quality and acceptability by trait. For every trait, expected quality was significantly positively associated with acceptability.*

| Trait | Pearson R | t (df=444) | 95% CI | p |
| --- | --- | --- | --- | --- |
| Aggression | 0.61 | 16.18 | [0.55, 0.66] | <.001 |
| Benevolence | 0.59 | 15.203 | [0.52, 0.64] | <.001 |
| Bravery | 0.65 | 17.878 | [0.59, 0.7] | <.001 |
| Confidence | 0.58 | 14.965 | [0.51, 0.64] | <.001 |
| Dementia | 0.63 | 17.198 | [0.57, 0.68] | <.001 |
| Depression | 0.58 | 15.057 | [0.52, 0.64] | <.001 |
| Extraversion | 0.57 | 14.45 | [0.5, 0.63] | <.001 |
| Generosity | 0.58 | 14.831 | [0.51, 0.63] | <.001 |
| Honour | 0.62 | 16.491 | [0.56, 0.67] | <.001 |
| Humility | 0.48 | 11.599 | [0.41, 0.55] | <.001 |
| Integrity | 0.64 | 17.63 | [0.58, 0.69] | <.001 |
| Leadership | 0.62 | 16.573 | [0.56, 0.67] | <.001 |
| Loyalty | 0.61 | 16.031 | [0.54, 0.66] | <.001 |
| Orderliness | 0.60 | 15.73 | [0.54, 0.65] | <.001 |
| Racism | 0.62 | 16.476 | [0.55, 0.67] | <.001 |
| Sexism | 0.59 | 15.345 | [0.52, 0.65] | <.001 |
| Social Anxiety | 0.62 | 16.622 | [0.56, 0.67] | <.001 |

*Section 3. Extended Information about Study 2a and 2b*

*<Figure S1>*

*Figure S1.* Example of a Moral Profile for a participant who scored higher on Care and Loyalty and lower on Fairness and Authority than the median participant in the YourMorals project database

*Study 2a Results*

To assess the relationship between participants' actual profile prevalence and their perceived prevalence with education, gender and politics, a linear model was applied (formula: difference ~ 1 + education + gender + politics). As stated in the main text and Table S3, these results indicate that people perceived their profiles as less prevalent than they were. Furthermore, demographics (education, gender, politics) had no effect on the model. Additionally, we note that, the model's intercept was calculated corresponding to education = “College or other tertiary qualification”, gender = “Female” and politics = “Conservative”. 95% Confidence Intervals (CIs) and p-values were computed using the Wald approximation.

Table S3. Results from the Linear Model used to Analyze Study 2a

*The effects in the model of Study 2a predicting the difference between the participant’s actual and perceived profile prevalence. The intercept indicates that participants’ actual profile prevalence was significantly higher than their perceived profile prevalence.*

| Term | Estimate | t(487) | p value | 95% CI |
| --- | --- | --- | --- | --- |
| (Intercept) | 2.08 | 2.78 | 0.006 | [0.61, 3.54] |
| Education: Up to High school diploma | -0.56 | -0.84 | 0.403 | [-1.88, 0.76] |
| Education: Some college but no degree | -0.22 | -0.42 | 0.675 | [-1.24, 0.81] |
| Gender: Male | -0.40 | -0.70 | 0.487 | [-1.56, 0.75] |
| Gender: Non-binary / third gender | -0.08 | -0.07 | 0.948 | [-2.62, 2.45], |
| Politics: Liberal | -0.11 | -0.14 | 0.887 | [-1.59, 1.37] |
| Politics: Moderate | -0.65 | -0.76 | 0.446 | [-2.32, 1.02] |
| Politics: Other | -0.55 | -0.42 | 0.677 | [-3.12, 2.03] |

*Study 2b Results*

Study B differed from Study 2a in that it incentivized correct responding with a financial reward, however, the pattern of results was for-the-most-part very similar to that in Study 2a. The relationship between participants’ actual and perceived profile prevalence was assessed using a linear model (formula: difference ~ 1 + education + gender + politics). As stated in the main text and presented in Table S4 the results show that people perceived their profiles as unique. Here, there were also effects of gender and politics. Males showed greater difference scores than females and liberals showed smaller difference scores than conservatives. No other effects were significant. As for S3, we note that the model's intercept was calculated corresponding to education = “College or other tertiary qualification”, gender = “Female” and politics = “Conservative”. 95% Confidence Intervals (CIs) and p-values were computed using the Wald approximation.

Table S4. Results from the Linear Model used to Analyze Study 2b

*The effects in the model of Study 2b predicting the difference between the participant’s actual and perceived profile prevalence. The intercept indicates that participants’ actual profile prevalence was significantly higher than their perceived profile prevalence.*

| Term | Estimate | 95% CI | t(488) | p value |
| --- | --- | --- | --- | --- |
| (Intercept) | 5.17 | [3.44, 6.90] | 5.87 | <.001 |
| Education: Up to High school diploma | -1.33 | [-3.54, 0.89] | -3.54 | .239 |
| Education: Some college but no degree | .20 | [-1.55, 1.95] | .22 | .822 |
| Gender: Male | 1.45 | [.07, 2.83] | 2.06 | .040 |
| Gender: Non-binary / third gender | -.55 | [-4.69, 3.59] | -.26 | .795 |
| Politics: Liberal | -1.70 | [-3.38, -0.02] | -1.99 | .047 |
| Politics: Moderate | .70 | [-1.26, 2.67] | .70 | .482 |
| Politics: Other | 4.29e-03 | [-3.66, 3.66] | 2.30e-03 | .998 |

*S4. Extended Information about Study 3 Method and Results*

*Study 3 Method*

<Figure_S2>

Figure S2. *Description of a moral profile provided to participants in Study 3. This image was presented alongside text: “Please read the following information carefully before moving to the next page. In the near future Artificial Intelligence may be used to generate moral profiles. A person's moral profile is a stable set of moral preferences and judgments. For example, the absolute and relative importance a person places on bravery, humility, generosity, honor, loyalty, benevolence and integrity. It may also incorporate a person's stance on issues like racism, sexism, and aggression. Some profiles are more common than others, we are interested in how you view the relationship between the quality of AI generated profiles and the prevalence of those profiles. That is, whether you think AI will do a better job at generating accurate profiles for people with common/typical profiles OR for people with rare/unique profiles.”*

*Section 4: Study 3 Results*

Study 3 replicated the findings of Study 1 for moral characteristics, showing a moderate to strong relationship between ratings of quality and acceptability. We fitted a linear mixed model (estimated using REML and nloptwrap optimizer) to predict acceptability ratings from expected quality ratings (formula: acceptability ~ quality). The model included trait and participant as random effects. As observed in Study 1, the effect of expected quality on acceptability was statistically significant and positive (beta = 0.47, 95% CI [0.45, 0.49], t(5055) = 38.64, p < .001; Std. beta = 0.42, 95% CI [0.40, 0.45]).

Uniqueness neglect scores were significantly different from the midpoint of the scale, indicating that people believed AI would perform worse when used to assess unique moral profiles. This was confirmed by fitting a constant (intercept-only) linear model (estimated using OLS), formula: uniqueness neglect ~ 1. The model's intercept was at 16.98 (95% CI [16.39, 17.57], t(5059) = 56.06, p < .001).

The expected accuracy ratings for AI-based assessments of the participants' own profiles (M= 47.76 of 100, SD=24.96) were associated with their uniqueness neglect scores. We fitted a linear model (estimated using OLS) to predict expected accuracy with unique neglect scores (formula: accuracy ~ uniqueness neglect). The effect of uniqueness neglect was statistically significant and negative (beta = -0.13, 95% CI [-0.17, -0.10], t(5058) = -8.32, p < .001; Std. beta = -0.12, 95% CI [-0.14, -0.09]). As expected, this indicated that the higher a person’s perception that AI does worse with a more unique profile, the lower they expect the accuracy of AI-based measures of their own profile.

The relationship between expected accuracy for one’s own profile and uniqueness neglect was also assessed separately for people who scored below the midpoint on the uniqueness neglect scale – indicating they did not believe AI assessments would be worse for unique profiles – and those who scored above the midpoint on the uniqueness neglect scale. We fitted two linear models (estimated using OLS) to predict expected accuracy with uniqueness neglect (formula: accuracy ~ uniqueness neglect). For those who did not show uniqueness neglect (<midpoint), there was no relationship between their uniqueness neglect score and their ratings of how well AI would do with their own profile; beta = 0.07, 95% CI [-0.06, 0.19], t(1038) = 1.08, p = 0.282; Std. beta = 0.03, 95% CI [-0.03, 0.09]. However, for those showing uniqueness neglect (>=midpoint), there was a significant negative relationship between their uniqueness neglect scores and their own profile accuracy rating; beta = -0.18, 95% CI [-0.23, -0.12], t(4018) = -6.42, p < .001; Std. beta = -0.10, 95% CI [-0.13, -0.07]. See Figure 3.

### Extended Data

*<Figure E1>*

Figure E1. *Data points reflect response from each participant on the acceptability and expected quality of AI-based scoring of 17 moral (positive and negative), mental health, and other traits. Lines reflect linear model with shaded 95% confidence intervals. Note: participants could only respond with integers (1-5), however, to avoid excessive overlap we have jittered the points (maximum of .1) around the integers points.*
