# Supplementary figures and images for: Humans feel too special for machines to score their morals
Source: PNAS Nexus. 2023 May 29;2(6):pgad179. doi: 10.1093/pnasnexus/pgad179 (PMC10266524; doi:10.1093/pnasnexus/pgad179)

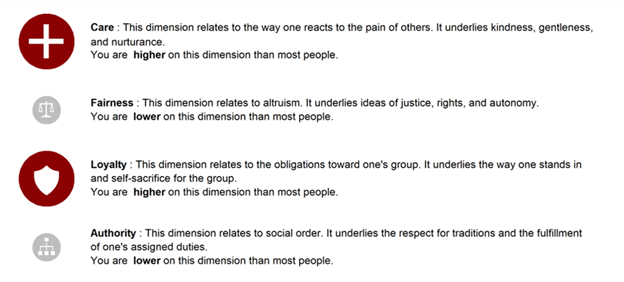

Supplement: pgad179_Supplementary_Data [file pgad179_supplementary_data.zip › PNASNEXUS-PNASNEXUS-2022-01256R-s01.tif]

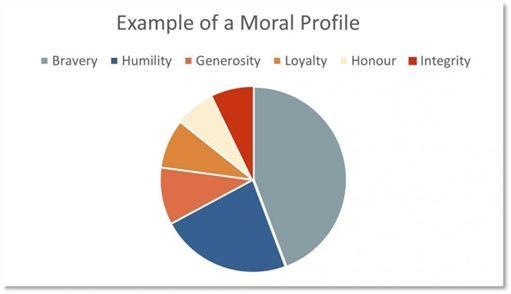

Supplement: pgad179_Supplementary_Data [file pgad179_supplementary_data.zip › PNASNEXUS-PNASNEXUS-2022-01256R-s02.tif]
